# 19p loss is significantly enriched in older age neuroblastoma patients and correlates with poor prognosis

**DOI:** 10.1038/s41525-020-0125-4

**Published:** 2020-04-15

**Authors:** Vito Alessandro Lasorsa, Flora Cimmino, Marzia Ognibene, Katia Mazzocco, Giovanni Erminio, Martina Morini, Massimo Conte, Achille Iolascon, Annalisa Pezzolo, Mario Capasso

**Affiliations:** 1 Dipartimento di Medicina Molecolare e Biotecnologie Mediche, Università degli Studi di Napoli Federico II, Napoli, Italy; 2 CEINGE Biotecnologie Avanzate, Napoli, Italy; 3 Laboratorio Cellule Staminali Post Natali e Terapie Cellulari, IRCCS Istituto Giannina Gaslini, Genova, Italy; 4 UOC Anatomia Patologica, IRCCS Istituto Giannina Gaslini, Genova, Italy; 5 Epidemiologia e Biostatistica IRCCS Istituto Giannina Gaslini, Genova, Italy; 6 Laboratorio di Biologia Molecolare, IRCCS Istituto Giannina Gaslini, Genova, Italy; 7 UOC Oncologia, IRCCS Istituto Giannina Gaslini, Genova, Italy; 8 IRCSS SDN, Napoli, Italy

**Keywords:** Prognostic markers, Cancer genomics

## Abstract

Genomic aberrations of neuroblastoma occurring in late childhood and adolescence are still understudied. Publicly available DNA copy number profiles of 556 tumors (discovery set) and of 208 tumors obtained by array-CGH assay (validation set) were used to test if 19p loss is significantly over-represented in children and adolescents with neuroblastoma. The 19p loss occurrence was separately tested within different age groups in the discovery and validation set and the resulting *P* values were combined by meta-analysis and corrected by Bonferroni’s method. In both sets, 19p loss was associated with older age at diagnosis. Particularly, the lowest age group significantly associated with 19p loss (discovery set: 20%; validation set: 35%) was 6 years. The 19p loss correlated with inferior overall survival in patients over 6 years of age. Relevant tumor suppressor genes (*KEAP1*, *DNM2*, *SMARCA4, SLC44A2* and *CDKN2D)* and microRNAs (miR-181c, miR-27a, and mirR-199a-1) are located in the genomic region involved in 19p loss. Downregulation of *DNM2*, *SLC44A2* and *CDKN2D* was associated with poor patient outcome and older age. Among the recurrent NB chromosomal aberrations, only 1q gain was enriched in patients older than 6, and its presence was mutually exclusive with respect to 19p loss. Our data demonstrate that 19p loss is a genomic biomarker of NB diagnosed in older children that can predict clinical outcome.

## Introduction

Neuroblastoma (NB) is the most frequent type of malignancy in the first year of life, but it rarely occurs after age 6 years and it is exceptionally unusual among adolescents^1^. Conte et al. suggested that along with the well-known favorable behavior of NB in children younger than 1 year, patients aged between 6 and 10 years might have a more indolent course but a worse prognosis^2^. Other studies reported that NB in late childhood and adolescence have unique biology characterized by an indolent disease course with long-term relapses and fatal outcomes, despite the presence of very few unfavorable known biologic markers^2–5^.

Genome-wide association studies, high-throughput sequencing and microarray gene expression-based studies have identified multiple genetic changes that characterize NB - both hereditable and somatically acquired^6–11^. Genetic alterations occurring in non-coding DNA such as *TERT* rearrangements^12^ and point mutations in regulatory elements of transcription factor binding sites^13^ also contribute to NB development. However, several recurrent segmental chromosomal alterations (SCA) have been demonstrated to better discriminate between low-risk and high-risk patients with fatal outcomes^10,11^. Unfortunately, in children and adolescents, the SCAs and their prognostic role remains little investigated. We thus reasoned that other, still unknown, biologic mechanisms might be involved in the natural history of NB in older age patients. Deletions of chromosome 19p have been previously reported in older age NB patients. Two studies reported 19p loss in 8 out of 21 adolescent patients^4,14^ and one study in 13 out of 86 patients older than 5 years^15^. However, to date, no large cohort has been investigated to verify if the presence of 19p loss is a chance finding, or if it significantly correlates with the occurrence of NB after a certain age. Moreover, no study has tested the specific clinical significance of 19p loss and its co-occurrence with other known SCAs.

Here, we evaluated the association of 19p loss with age at diagnosis using different age groups in a large, public, genomic dataset (*N* = 556) and our unpublished array-CGH (comparative genomic hybridization) dataset of Italian cases (*N* = 208). We observed significant enrichment of 19p loss in older age patients (over 6 years of age) and a significant correlation between 19p loss and poor survival rates among patients older than 6 years. In this sub-set of older patients, 1q gain was also over-represented but did not co-occur with 19p loss.

## Results

### Chromosome 19p loss is enriched in NBs diagnosed after 6 years

**Fig. 1:**
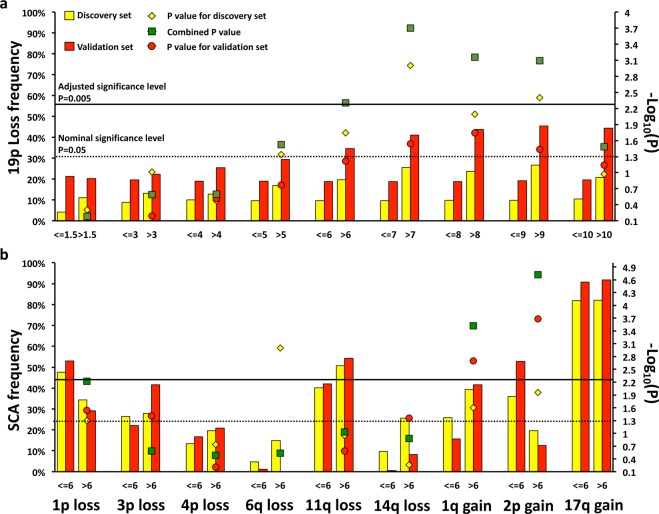
Prevalence of 19p loss in different age groups. The bar plot shows the prevalence of **a** 19p loss at different ages at diagnosis and of **b** diverse SCA at 6 years age group in two independent datasets. The statistically significant difference of 19p loss frequency is also shown.

To identify the age group significantly associated with the presence of 19p loss, we first tested the association in the discovery and in the validation sets separately (Supplementary Table 1). Overall, the 19p loss frequency was higher in the discovery (11%) when compared to validation set (21%) probably due to the different sample size and platforms used in the two datasets (Fig. 1a and Supplementary Table 2).

Then, we combined the results by meta-analysis. In the combined analysis, we found that 19p loss was significantly enriched in patients with age at diagnosis greater than 6, 7, 8 and 9 years (*P*_corrected_ < 0.005) (Fig. 1a, Supplementary Table 2). We also observed an association between 19p loss and age greater than 10 years (9 out of 33 cases), but it was not statistically significant after *P*-value correction (*P*_combined_ = 0.03; *P*_corrected_ = 0.30). Indeed, the lowest age significantly associated, in the combined analysis, with 19p loss was greater than 6 years. Moreover, 6 years of age was the lowest cut-off that significantly associated with 19p loss when separate analyses were performed in the two datasets (Supplementary Table 2). The frequency of 19p loss in the group with age greater than 6 years was 20% for discovery set and 35% for validation set. By contrast, the group with age <= 6 years showed a frequency of 19p loss of 10 and 19%, for discovery and validation sets, respectively. Therefore, based on these results, the following analyses were restricted to the sub-set of patients older than 6 years.

### Tumor-suppressor genes and microRNAs located in 19 deleted region

**Fig. 2:**
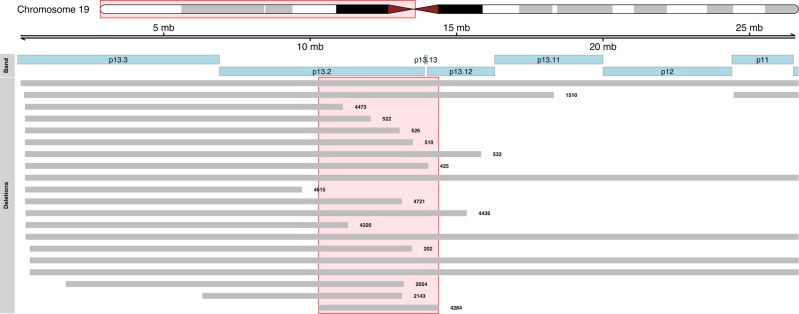
Deleted regions of 19p in patients older than 6 years. Genomic view of chromosome 19p (hg19 genome assembly). Gray tracks report the deleted region found in patients older than 6 years. The minimally deleted region (track 4284), is indicated by the red box.

By combining the two datasets, we found that the minimally deleted region (MDR), defined by the patient 4284 (4098 Kb), occurred in 9 out of 21 cases (43%) and contained 156 genes (Fig. 2 and Supplementary Table 3) including 141 protein-coding genes. *KEAP1*, *DNM2*, *SMARCA4* were annotated as tumor suppressor genes in the Cancer Gene Census database. A case with 6.76 Mb loss (from 19p13.3 to p13.13) encompassing the gene *SMARCA4* had also a stop gain germline mutation in the same gene (data from whole-exome sequencing). Downregulation of 27 genes in RNAseq data from 498 tumors was associated with decreased overall survival (OS) and/or event-free survival (EFS) (Supplementary Table 4). Among these genes, expression of *SLC44A2* and *CDKN2D* was confirmed to be correlated with decreased OS and/or EFS in two independent microarray gene expression datasets (Supplementary Table 4). Low expression of *SLC44A2*, *CDKN2D, and DNM2* was also associated with unfavorable clinical markers such as Stage 4 disease, *MYCN* amplification (MNA) (Supplementary Fig. 1), and age at diagnosis (>1.5, 6, 7, 8, 9, and 10 years) (Supplementary Fig. 2). In the same MDR, we found the micro(mi)RNA-181c which functions as tumor-suppressor in NB^16,17^. Other well-studied tumor suppressor miRNAs were miR-27a and mirR-199a-1^18,19^.

### 19p loss is an independent SCA in NBs diagnosed after 6 years

We verified if other recurrent SCA in NB^11^ were enriched in patients with age greater than 6 years. We found that 1p loss and 2p gain were under-represented, whereas 1q gain was over-represented in both datasets (Fig. 1b, Supplementary Table 5). However, 1q gain, 1p loss, and 2p gain did not co-occur with 19p loss, and 1q gain and 19p loss seemed to be mutually exclusive (Supplementary Fig. 3).

### Prognostic implications of 19p loss

**Fig. 3:**
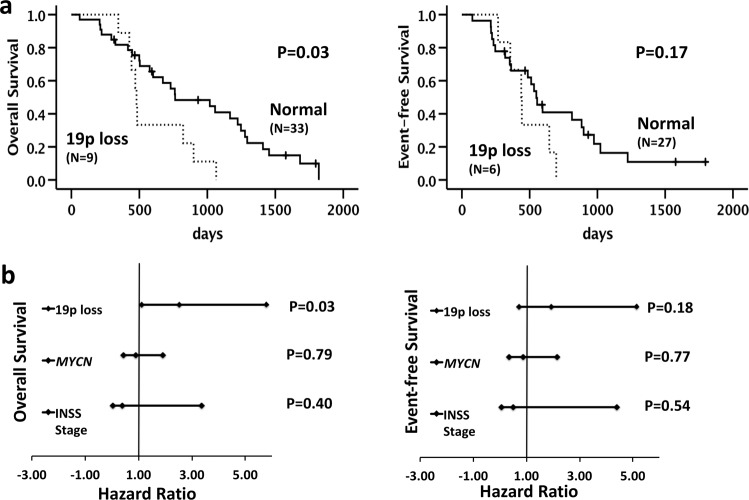
Survival analysis. **a** OS and EFS using Kaplan–Meier analysis show difference in the survival between NBs with and without 19p loss. **b** Forest plots represent the multivariate models of OS and EFS including potential prognostic factors as *MYCN* status (*MYCN* amplified versus MYCN non-amplified), stage (4 versus 1, 2, 3, 4S), and 19p loss (normal *versus* affected). The hazard ratio and the associated *p* values were calculated using Cox proportional hazard analysis.

In the discovery set, including only patients older than 6 years, 19p loss was found to be a significant marker for OS. Five-year OS was 5% ± 7% and 14% ± 6% for 11 patients with and 35 patients without 19p loss, respectively (Fig. 3a, *P* = 0.03). A non-significant effect of 19p loss on EFS was observed (Fig. 3a). The presence of other known SCAs was not significantly correlated with OS and EFS (Supplementary Table 6). Multivariate analysis including risk factors such as *MYCN* status and INSS stage confirmed 19p loss as independent marker for OS (Fig. 3b). These results were not confirmed when the same analyses were performed using data from the validation set (data not shown) probably due to the smaller sample size (only 21 samples were analyzed).

## Discussion

Recent literature has highlighted that NB diagnosed in children and adolescents may exhibit unusual genomic aberrations with indolent but often fatal clinical course^2–5^. Nevertheless, tumor biology of older NB patients is little investigated and still unknown, as NB is a rare disease, making it challenging to collect enough tumor material and data for a sufficiently powered study. Deletions of 19p loss have been observed in older children^4,14,15^. However, previous studies have not provided an in-depth analysis of this SCA in large and independent sets of patients. Therefore, its relevance to tumor biology and clinical management of NB remains unclear. Until now, a comprehensive association analysis of 19p loss and age at diagnosis, its prognostic value and co-occurrence with other known SCAs, and a description of potential cancer genes in its MDR were still lacking. Here, using two large datasets of copy number variant profiles, we have demonstrated that the frequency of 19p loss is significantly enriched in patients with age at onset over 6 years. Notably, among the already known and frequently detected SCA in NB, only 1q gain resulted over-represented in older patients (age > 6 years), and did not occur with 19p loss whereas, as already observed by Mossè et al.^5^ in children > = 5 to <10 years and > = 10 years, 2p gain and 1p loss were under-represented. Together, these data further support the hypothesis that patients with age at diagnosis between 5 and 10 years might represent a “different” group of patients with peculiar clinical and genetic features^2^. However, additional studies are needed to verify if our proposed age threshold (6 years) is the most suitable to define a sub-group of older patients with different biological and clinical characteristics with respect to younger patients. Since the overall 19p loss frequency resulted quite different between the two datasets, further studies are warranted to determine its exact prevalence among older children.

We found some interesting genes in the MDR of 19p loss occurring in patients older than 6 years. *SMARCA4* is part of the SWI/SNF (mating type Switching defective/Sucrose Non-Fermenting) chromatin-remodeling complex. Germline mutations in *SMARCA4* are known to predispose to the development of the rhabdoid tumor predisposition syndrome^20^. Additionally, inactivating somatic mutations in *SMARCA4* have been reported in many cancer cell lines, including non-small cell lung cancer and small cell carcinoma of the ovary^21,22^. Recently, we and others have found *SMARCA4* somatic point mutations in NB, highlighting its role in the oncogenesis of this neuroblastic tumor^7,23^.

Another interesting gene that could be impacted by 19p loss is *CDKN2D*. The protein (p19-INK4d) coded by this gene belongs to the INK4 family of CDK4/6 kinase inhibitors together with p16-INK4a, p15-INK4b, and p18-INK4c. p19-INK4d plays a critical role on cell cycle regulation blocking G1-S transition by CDK4 inhibition^24^. We found that low expression of *CDKN2D* correlated with poor patient survival and known NB risk factors such as MNA, suggesting a tumor suppressor role. Accordingly, Dreidax et al. have experimentally demonstrated the tumor suppressive role of this gene in NB and have identified a region within the gene whose hypermethylation is associated with *CDKN2D* repression and MNA^25^. In our analysis, we found no co-occurrence between MNA and *CDKN2D* deletion, suggesting that diverse and exclusive genomic alterations may affect *CDKN2D* function in NB. In addition, another independent study suggested that *CDKN2D* gene functions as tumor-suppressor, demonstrating that deletion of *CDKN2D* resulted in spontaneous development of tumors in multiple murine organs and tissues^25^.

The *DNM2* gene is located on 19p13.2 and encodes for Dynamin 2, one of the subfamilies of GTP-binding proteins. Dynamin 2 plays a role in the regulation of neuron morphology, axon growth and formation of neuronal growth cones, and it is also involved in cytokinesis^26^. Heterozygous pathogenic variants in *DNM2* are associated with Charcot-Marie-Tooth neuropathy, a disorder of the peripheral nervous system (OMIM: 606482). These data suggest a potential role of *DNM2* in neuronal differentiation and maintenance; therefore, inactivating mutations in this gene may be responsible of altered neuroblast differentiation that can promote tumorigenesis.

Although the above-commented genes can be considered as good novel candidate cancer genes, their real involvement in NB tumorigenesis must be verified by appropriate biological studies.

In the MDR, we also found miRNAs that have been suggested to act as tumor-suppressors such as miR-181c^16,17^, miR-27a^27,28^, and mirR-199a-1^18,19^. Notably, low expression levels of the miR-181c were found in NB cancer tissues when compared with adjacent tissues, and associated with decreased proliferation of NB cell lines and unfavorable clinical parameters such as metastasis, poor tumor differentiation, and advanced stages^16^. Another study showed that miR-181c was downregulated in metastatic NB tissues compared with primary NB tissues and that acted as tumor suppressor by reducing *SMAD7* expression^17^.

The 19p loss could represent a novel independent marker of prognosis for older NB patients. Indeed, significant decrease of OS was observed in patients with age at diagnosis greater than 6 years and 19p loss. Nevertheless, statistical significance was not achieved for association with decreased EFS. Larger studies are warranted to investigate and validate the independent prognostic importance of this aberration.

Our results demonstrate that 19p loss represents a typical SCA of NB tumors arisen in older patients, that can be a useful prognostic marker in this rare sub-set of patients. In addition, this study demonstrates the utility of studying genetic changes in cancer diagnosed in older age pediatric patients to better predict patient outcome and develop specifically tailored treatment protocols.

## Methods

### Ethics statement

This study was approved by the Institutional Ethics Committee of Regione Liguria (Prot. n° IGG-NCA-AP-2016). Written informed consent was obtained from all patients or their legal guardians. The patient data were derived from Italian Neuroblastoma Registry (INBR) of AIEOP.

### Datasets

We used two large datasets of copy number profiles. A large and high quality cured public dataset of 556 high-risk NB tumors^29^ was used as “discovery set” (GEO accession: GSE103123) whereas our unpublished dataset of 208 NB tumors was used as “validation set” (Supplementary Table 1). Clinical information including age at diagnosis, disease stage overall (OS) and event-free survival (EFS) were available for both datasets^3^. For discovery set, we used the aberration calls deposited in Depuydt et al.^29^. For both datasets, segmental aberrations of chromosome arms were defined as gains and losses larger than 3 Mb, excluding whole chromosome aberrations.

### Association analysis of 19p loss and age at diagnosis

In order to identify if an optimal age threshold exists that defines a group of patients with statistically significant occurrence of 19p loss, association between age at diagnosis and 19p loss was evaluated. The cohort of children >18 months at diagnosis was repeatedly divided into two groups at all possible yearly points between 3 and 10 years, that is, 9 different cut-offs. These paired age groups (younger *vs*. older) were compared using two-sided Chi-square tests or two-sided Fisher exact tests in data with expected frequencies <5. The analysis was separately performed in discovery and validation set and the resulting significance values were combined (by Stouffer’s method) and corrected according to Bonferroni multiple comparison test.

### Primary NB tumors in validation set

A multi-institution retrospective series of primary tumors from 208 distinct NB patients of the Italian Association of Pediatric Hematology and Oncology (AIEOP) was analyzed. All the tumors were classified according to the International Neuroblastoma Staging System. NB tissues were obtained at the time of initial diagnosis before treatment. Tumor content was confirmed by review of hematoxylin and eosin stained tumor sections by the local pathologists. Tumor DNAs were extracted using the QIAamp DNA Extraction Kit (Qiagen, Hilden, Germany), according to the manufacturer’s instructions.

### Array-CGH analysis in validation set

All samples were profiled on CGH oligonucleotide array platform (Agilent Technologies, Santa Clara, California, USA) with a mean resolution of ~25 kb. Oligo-array data were analyzed with Genomic Workbench 7.0.40 software (Agilent). Chromosome positions were determined using GRCh37/hg19 (UCSC Genome Browser, http://genome.ucsc.edu, Feb. 2009 release). To make aberration calls, an aberration detection algorithm ADM-1 was used at threshold 10, and an aberration filter was set at 2 for the minimum number of probe region, and 1 for minimum absolute average log2 ratio to reduce false positives. The quality of the test was assessed on the strength of the QCmetrics values. Polymorphisms (http://projects.trag.ca/variation/) were not included because considered normal variants. As in discovery set, gains and losses were set at 0.2 and −0.3, respectively whereas amplification and homozygous deletions were set at 2 and −2, respectively.

### Statistical analysis

Combined *P* values were obtained by Stouffer’s method. Multiple testing correction relied on the Bonferroni’s Multiple comparison Test. An alpha of 0.05 was used as the cut-off for significance. Kaplan–Meier estimates were calculated with SPSS program. *P* values reported with Kaplan–Meier plots resulted from log-rank tests.

### Reporting summary

Further information on experimental design is available in the Nature Research Reporting Summary linked to this article.

## Supplementary information

Supplementary Information

Reporting Summary

## Data Availability

The data generated in this study have been deposited at the GEO database under the accession number: GSE145341.

## Code availability

The data and the results generated in this study were obtained without using any custom code or mathematical algorithm.
