# Supplementary material for: 19p loss is significantly enriched in older age neuroblastoma patients and correlates with poor prognosis
Source: NPJ Genom Med. 2020 Apr 15;5:18. doi: 10.1038/s41525-020-0125-4 (PMC7160145; doi:10.1038/s41525-020-0125-4)
Supplement: Supplementary file 1 — Supplementary Information [file 41525_2020_125_MOESM1_ESM.pdf]

SUPPLEMENTARY INFORMATION

19p loss is significantly enriched in older age neuroblastoma patients and correlates with poor prognosis

Vito Alessandro Lasorsa[1,2], Flora Cimmino[1,2], Marzia Ognibene[3], Katia Mazzocco,[4] Giovanni Erminio[5], Martina Morini[6], Massimo Conte[7], Achille Iolascon[1,2], ^*Annalisa Pezzolo[3], ^*Mario Capasso[1,2,8]

[1]Dipartimento di Medicina Molecolare e Biotecnologie Mediche, Università degli Studi di Napoli Federico II, Napoli, Italy

[2]CEINGE Biotecnologie Avanzate, Napoli, Italy

[3]Laboratorio Cellule Staminali Post Natali e Terapie Cellulari, IRCCS Istituto, Italy Giannina Gaslini Genova,

[4]UOC Anatomia Patologica, IRCCS Istituto Giannina Gaslini Genova, Italy

[5]Epidemiologia e Biostatistica IRCCS Istituto Giannina Gaslini Genova, Italy

[6]Laboratorio di Biologia Molecolare, IRCCS Istituto Giannina Gaslini Genova Italy

[7]UOC Oncologia, IRCCS Istituto Gaslini, Genova, Italy.

[8]IRCSS SDN, Napoli

^These authors equally contributed to this work

***Corresponding Authors Information**:

Mario Capasso, Università degli Studi di Napoli "Federico II", via Gaetano Salvatore 486, 80145 Napoli; e-mail: mario.capasso@unina.it

Annalisa Pezzolo, IRCCS Istituto Gaslini, Via Gerolamo Gaslini 3, 16147 Genova, Italy. e-mail: annalisapezzolo@gaslini.org

SUPPLEMENTARY FIGURES

Supplementary Figure 1. Association between clinical markers and *SLC44A2*, *CDKN2D* and *DNM2* in 498 NB tumors profiled by RNAseq (GSE62564). P values are obtained by using Manny-Whitney test.

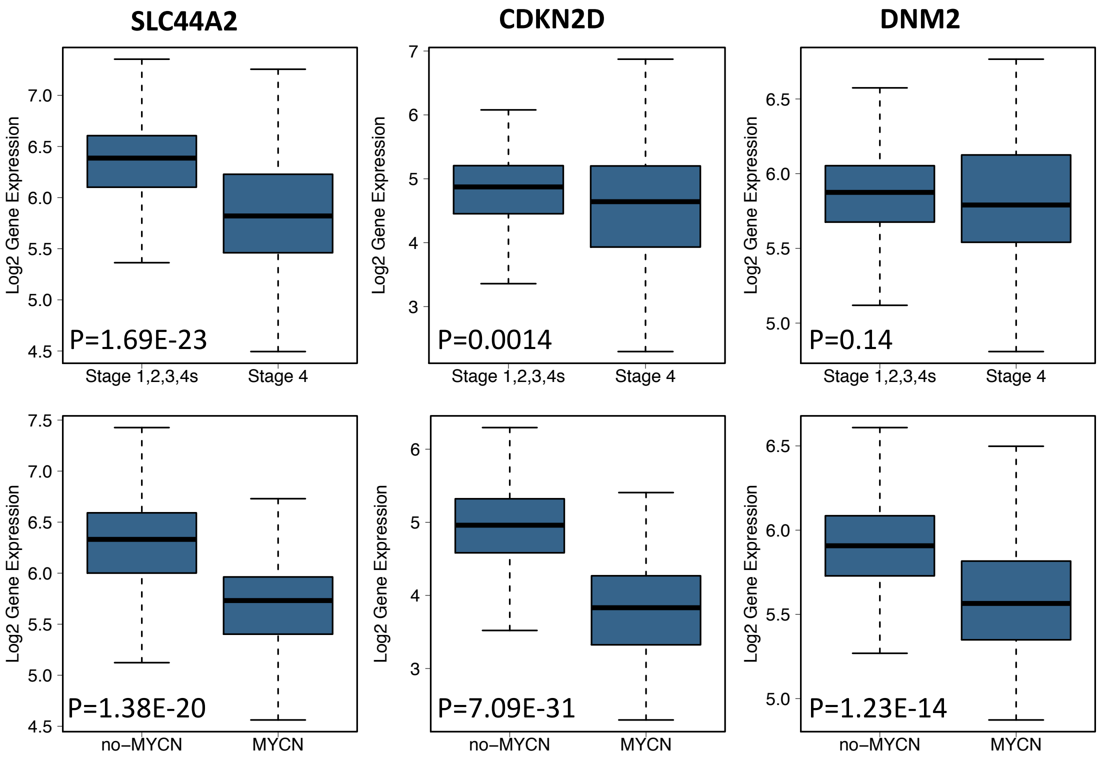

Supplementary Figure 2. Association between age thresholds and *SLC44A2*, *CDKN2D* and *DNM2* in 498 NB tumors profiled by RNAseq (GSE62564). P values are obtained by using Manny-Whitney test.

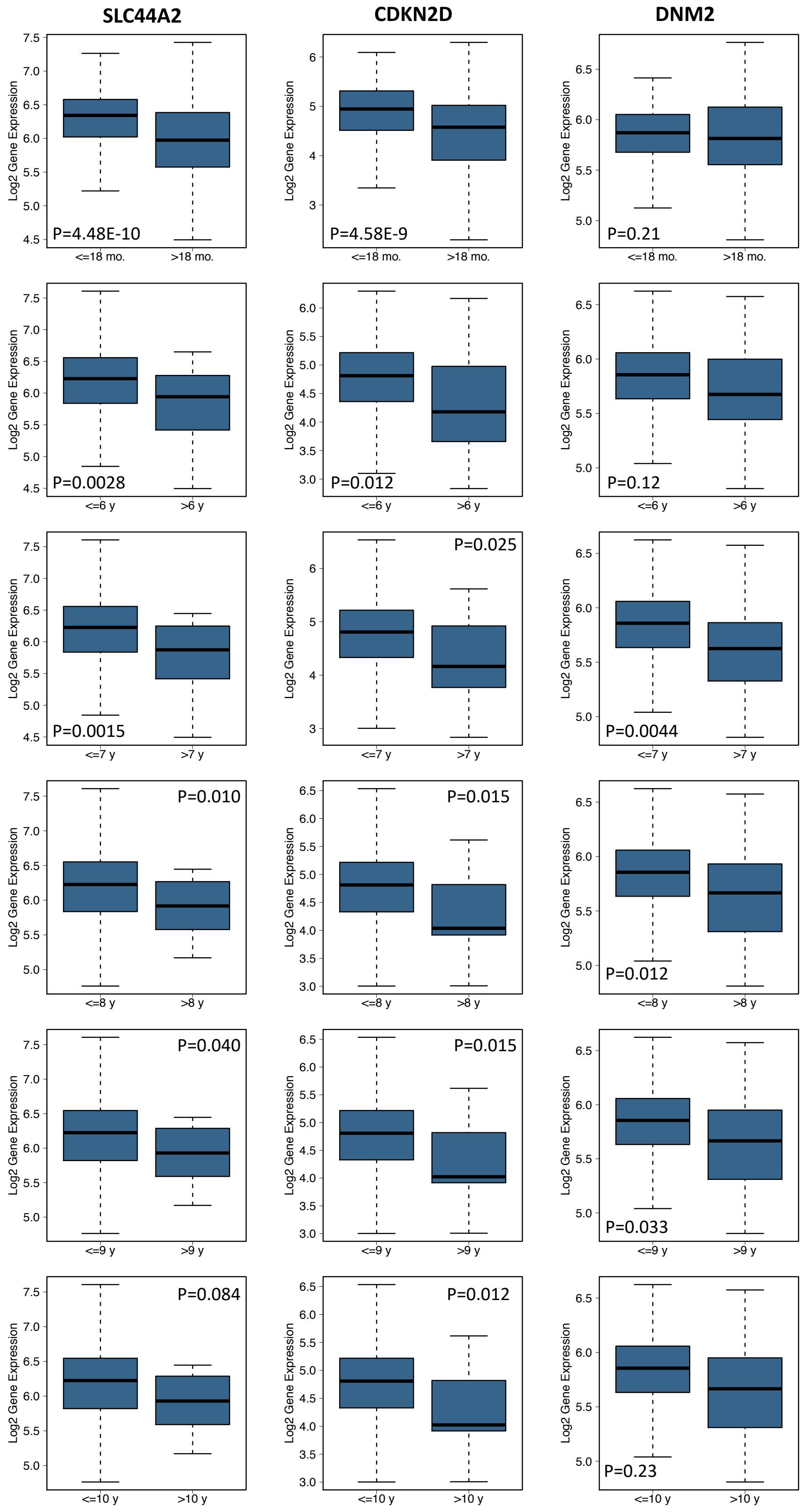

Supplementary Figure 3. Plot showing mutual exclusivity and co-occurrence among 19p loss and other common NB genomic aberrations. Chi-square test: *P=0.17 and ^P=0.034.

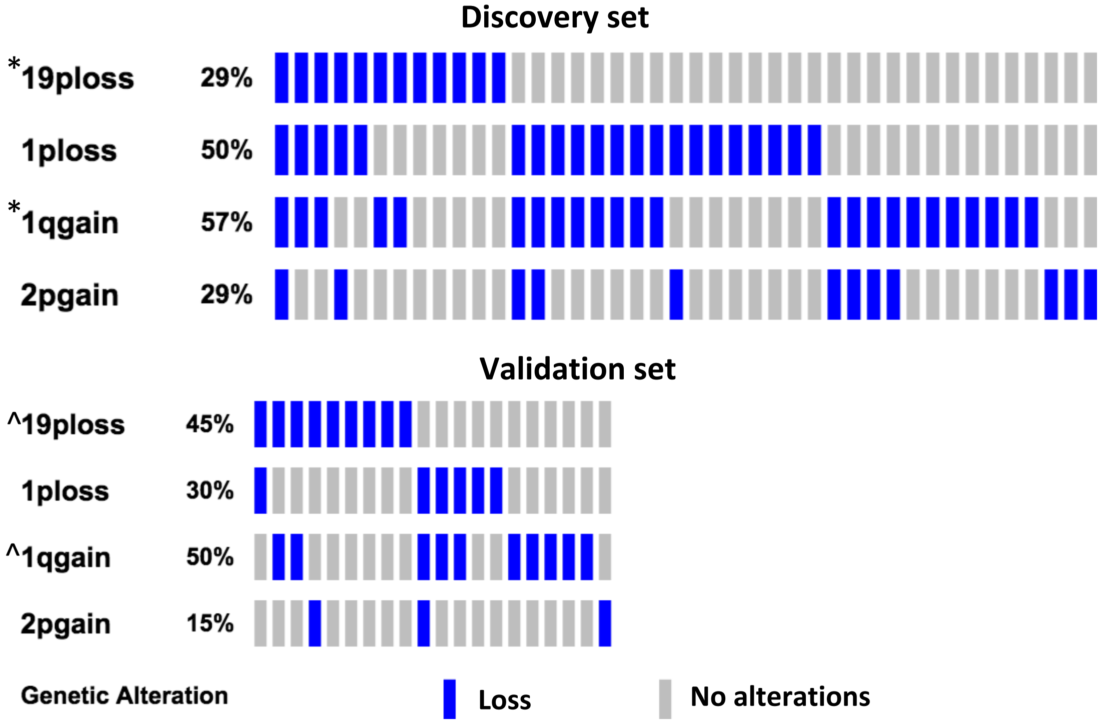

SUPPLEMENTARY TABLES

Supplementary Table 1. Clinical characteristics of Italian Neuroblastoma patients (N=208).

Variable	Number (%)
**Age**	
≥ 18 m	80 (38)
< 18 m	128 (62)
**INSS Stage**	
4	98 (48)
1,2,3,4s	104 (52)
Unknown	6
**MYCN**	
Amplified	48 (24)
Not amplified	154 (76)
Unknown	6

The number of patients is expressed as Value (percentage).

Supplementary Table 2. Prevalence of 19p loss in NB patients according to different cut-off ages at diagnosis.

	Discovery set (N=554)			Validation set (N=208)				
Cut-off age	19p normal	19 loss	P	19p normal	19 loss	P	P_combined	P_corrected
<=1.5 years	23 (5%)	1 (2%)		63 (38%)	17 (40%)			
>1.5 years	471 (95%)	59 (98%)	0.499	102 (62%)	26 (61%)	0.871	0.653	1.000
<=3 years	270 (55%)	26 (43%)		106 (64%)	26 (61%)			
>3 years	224 (45%)	34 (57%)	0.097	59 (36%)	17 (40%)	0.647	0.257	1.000
<=4 years	351 (71%)	39 (65%)		124 (75%)	29 (67%)			
>4 years	143 (29%)	21 (35%)	0.332	41 (25%)	14 (33%)	0.307	0.253	1.000
<=5 years	420 (85%)	45 (75%)		141 (86%)	33 (77%)			
>5 years	74 (15%)	15 (25%)	0.046	24 (15%)	10 (23%)	0.169	0.030	0.270
**<=6 years**	**445 (90%)**	**48 (80%)**		**148 (90%)**	**34 (79%)**			
**>6 years**	**49 (10%)**	**12 (20%)**	**0.018**	**17 (10%)**	**9 (21%)**	**0.061**	**0.005**	**0.044**
**<=7 years**	**462 (94%)**	**49 (82%)**		**155 (94%)**	**36 (84%)**			
**>7 years**	**32 (7%)**	**11 (18%)**	**0.001**	**10 (6%)**	**7 (16%)**	**0.029**	**0.0002**	**0.002**
**<=8 years**	**465 (94%)**	**51 (85%)**		**156 (95%)**	**36 (84%)**			
**>8 years**	**29 (6%)**	**9 (15%)**	**0.008**	**9 (6%)**	**7 (16%)**	**0.018**	**0.0007**	**0.006**
**<=9 years**	**472 (96%)**	**52 (87%)**		**159 (96%)**	**38 (88%)**			
**>9 years**	**22 (5%)**	**8 (13%)**	**0.004**	**6 (4%)**	**5 (12%)**	**0.052**	**0.0008**	**0.007**
<=10 years	475 (96%)	55 (92%)		160 (97%)	39 (91%)			
>10 years	19 (4%)	5 (8%)	0.166	5 (3%)	4 (9%)	0.09	0.033	0.297

P_combined: Stouffer method.
P_corrected: Bonferroni correction.
In bold the significant results.

Supplementary Table 3. Genes in the MDR of 19p deletions.

Gene	Is TSG	Gene	Is TSG	Gene	Is TSG
DNMT1	.	ZNF844	.	ZNF627	.
S1PR2	.	ZNF20	.	HNRNPA1P10	.
MIR4322	.	ZNF625	.	ZNF833P	.
MRPL4	.	ZNF136	.	ZNF823	.
ICAM1	.	LOC100289333	.	ZNF441	.
ICAM4	.	ZNF44	.	ZNF491	.
ICAM5	.	ZNF563	.	ZNF440	.
ZGLP1	.	ZNF442	.	ZNF439	.
RAVER1	.	ZNF799	.	ZNF69	.
ICAM3	.	ZNF443	.	ZNF700	.
TYK2	.	ZNF709	.	ZNF763	.
CDC37	.	ZNF564	.	ZNF433	.
MIR1181	.	ZNF490	.	ZNF878	.
PDE4A	.	ZNF791	.	TRIR	.
KEAP1	Yes	MAN2B1	.	FDX2	.
S1PR5	.	WDR83	.	ILF3-DT	.
ATG4D	.	WDR83OS	.	ZNF433-AS1	.
MIR1238	.	DHPS	.	GNG14	.
KRI1	.	FBXW9	.	ZNF788P	.
CDKN2D	.	TNPO2	.	MIR181D	.
AP1M2	.	SNORD135	.	NANOS3	.
SLC44A2	.	SNORD41	.	C19orf57	.
ILF3	.	ASNA1	.	CC2D1A	.
QTRT1	.	BEST2	.	PODNL1	.
DNM2	Yes	HOOK2	.	DCAF15	.
MIR638	.	MIR5684	.	RFX1	.
MIR4748	.	JUNB	.	RLN3	.
MIR199A1	.	PRDX2	.	IL27RA	.
MIR6793	.	RNASEH2A	.	PALM3	.
TMED1	.	RTBDN	.	MISP3	.
C19orf38	.	MAST1	.	MIR1199	.
CARM1	.	MIR6794	.	C19orf67	.
YIPF2	.	DNASE2	.	SAMD1	.
TIMM29	.	KLF1	.	PRKACA	.
SMARCA4	Yes	GCDH	.	ASF1B	.
LDLR	.	SYCE2	.	LOC100507373	.
MIR6886	.	MIR5695	.	ADGRL1	.
SPC24	.	FARSA	.	ELAVL3	.
KANK2	.	CALR	.	ZNF653	.
DOCK6	.	MIR6515	.	MIR7974	.
LOC105372273	.	RAD23A	.	ECSIT	.
ANGPTL8	.	GADD45GIP1	.	CNN1	.
TSPAN16	.	DAND5	.	ELOF1	.
RAB3D	.	NFIX	.	ACP5	.
TMEM205	.	LYL1	.	C19orf53	.
CCDC159	.	TRMT1	.	ZSWIM4	.
PLPPR2	.	NACC1	.	LOC284454	.
SWSAP1	.	STX10	.	MIR24-2	.
EPOR	.	IER2	.	MIR27A	.
RGL3	.	CACNA1A	.	MIR23A	.
CCDC151	.	CCDC130	.	MIR181C	.
PRKCSH	.	MRI1	.		

TSG: Tumor Suppressor Gene.

Supplementary Table 4. Effect of low expression of genes located at MDR on survival of NB patients according to different datasets in R2 database.

Dataset	Survival	PALM3	SLC44A2	RTBDN	CC2D1A	CDKN2D	CCDC159	ICAM1	IL27RA	MAST1	PRKACA	TNPO2	LOC284454	ZNF441	C19ORF57	MIR24-2	RGL3	PODNL1	DOCK6	MIR27A	C19ORF38	EPOR	ACP5	DNASE2	ICAM5	S1PR5	TYK2	LOC100507373	Source
Tumor Neuroblastoma - SEQC - 498 - RPM - seqcnb1	EFS	1.00E-10	4.56E-10	4.49E-09	2.96E-08	5.28E-08	1.85E-07	4.58E-07	8.94E-06	1.88E-05	2.07E-05	1.69E-04	4.61E-04	1.53E-03	2.54E-03	4.84E-03	4.91E-03	4.95E-03	5.59E-03	1.01E-02	1.12E-02	2.39E-02	NS	NS	NS	NS	NS	NS	GEO ID: GSE62564
	OS	1.78E-17	6.26E-15	9.61E-13	2.67E-10	1.55E-12	1.13E-10	8.60E-17	8.88E-08	7.80E-05	1.49E-07	7.72E-03	2.23E-09	NS	NS	NS	NS	7.94E-04	2.47E-03	NS	NS	NS	6.74E-08	1.32E-05	1.89E-05	8.26E-03	1.07E-02	3.20E-02	
TARGET - Asgharzadeh 249 - custom - huex10t	EFS	NA	3.60E-02	NS	NS	NS	NS	NS	NS	NS	NS	NS	NA	NS	NS	NA	NS	NS	NS	NA	NA	NS	NS	NS	NS	NS	NS	NS	TARGET website data matrix
	OS	NA	2.00E-02	NS	NS	NS	NS	NS	NS	NS	NS	NS	NA	NS	NS	NA	NS	NS	NS	NA	NA	NS	NS	NS	NS	NS	NS	NS	
Tumor Neuroblastoma non MYCN amplified - Seeger - 102 - MAS5.0 - u133a	relapse free	NA	NA	NA	NS	1.50E-07	NA	NS	NS	1.30E-03	NS	1.50E-02	NA	NA	NS	NA	5.20E-03	NS	NS	NA	NA	1.70E-02	NS	9.10E-03	1.10E-02	3.60E-02	NS	NA	GEO ID: GSE3446
Tumor Neuroblastoma Versteeg 88 - MAS5.0 - u133p2	relapse free	NS	NS	NS	NS	1.20E-02	NS	NS	NS	NS	NS	NS	NA	NS	NS	NA	NS	NS	NS	NA	NA	NS	NS	NS	NS	NS	NS	NA	GEO ID: GSE16476
	OS	2.1E-03	NS	NS	NS	9.20E-05	1.7E-02	NS	NS	NS	NS	NS	NA	NS	NS	NA	NS	NS	NS	NA	NA	NS	NS	NS	NS	NS	NS	NA	
Tumor Neuroblastoma Primary - NRC - 283 - rma_sketch (bc) - huex10t	progression free	NA	2.60E-02	NS	NS	NS	NS	NS	NS	NS	NS	NS	NA	NS	NS	NA	NS	NS	NS	NA	NA	NS	NS	NS	NS	NS	NS	NS	GEO ID: GSE85047
	OS	NA	2.40E-04	NS	NS	NS	NS	NS	NS	NS	NS	NS	NA	NS	NS	NA	NS	NS	NS	NA	NA	NS	NS	NS	NS	NS	NS	NS	

NS: not significant.

NA: not available.

Dataset of 498 samples is considered as discovery set and the obtained p-values are corrected according to Bonferroni method.

Supplementary Table 5. Prevalence of recurrent SCAs in NB patients according to different cut-off ages at diagnosis.

	**Discovery set (N=554)**			**Validation set (N=208)**				
Cut-off age	<=6 years	>6 years	P	<=6 years	>6 years	P	P_combined	P_corrected
**1p**	**258 (52%)**	**40 (66%)**	**0.05**	**76 (47%)**	**17 (71%)**	**0.029**	**0.006**	**0.054**
**1p loss**	**235 (48%)**	**21 (34%)**		**86 (53%)**	**7 (29%)**			
3p	363 (74%)	44 (72%)	0.802	126 (78%)	14 (58%)	0.039	0.259	2.331
3p loss	130 (26%)	17 (28%)		36 (22%)	10 (42%)			
4p	427 (87%)	49 (80%)	0.183	135 (83%)	19 (79%)	0.614	0.332	2.988
4p loss	66 (13%)	12 (20%)		27 (17%)	5 (21%)			
6q	470 (95%)	52 (85%)	0.001	160 (99%)	24 (100%)	1	0.294	2.646
6q loss	23 (5%)	9 (15%)		2 (1%)	0 (0%)			
11q	295 (60%)	30 (9%)	0.111	94 (58%)	11 (46%)	0.261	0.094	0.846
11q loss	198 (40%)	31 (51%)		68 (42%)	13 (54%)			
14q	448 (91%)	54 (88%)	0.553	161 (99%)	22 (92%)	0.044	0.133	1.197
14q loss	45 (9%)	7 (12%)		1 (1%)	2 (8%)			
**1q**	**366 (74%)**	**37 (61%)**	**0.025**	**145 (84%)**	**14 (58%)**	**0.002**	**0.0003**	**0.0027**
**1q gain**	**127 (26%)**	**24 (39%)**		**27 (16%)**	**10 (42%)**			
**2p**	**315 (64%)**	**49 (80%)**	**0.011**	**81 (47%)**	**21 (87%)**	**0.0002**	**0.000019**	**0.00017**
**2p loss**	**178 (36%)**	**12 (20%)**		**91 (53%)**	**3 (13%)**			
17q	89 (18%)	11 (18%)	0.997	16 (9%)	2 (8%)	0.878	0.99	8.91
17q gain	404 (82%)	50 (82%)		156 (91%)	22 (92%)			

P_combined: Stouffer method.
P_corrected: Bonferroni correction.
In bold the significant results.

Supplementary Table 6. OS and EFS analysis for known SCAs.

Dataset	Survival	1p loss	3p loss	4p loss	6q loss	11q loss	14q loss	1q gain	2p gain	17q gain
Discovery set (>6y)	EFS	6.20E-01	4.10E-01	2.80E-01	6.00E-01	8.00E-01	7.40E-01	4.80E-01	6.00E-01	2.30E-01
	OS	2.90E-01	2.60E-01	2.40E-01	9.60E-01	2.90E-01	8.60E-01	7.90E-01	2.20E-01	1.30E-01
